# Transcriptomic changes of *Legionella pneumophila* in water

**DOI:** 10.1186/s12864-015-1869-6

**Published:** 2015-08-26

**Authors:** Laam Li, Nilmini Mendis, Hana Trigui, Sébastien P. Faucher

**Affiliations:** Department of Natural Resource Sciences, Faculty of Agricultural and Environmental Sciences, McGill University, 21,111 Lakeshore Road, Ste-Anne-de-Bellevue, Montreal, QC H9X 3V9 Canada

**Keywords:** *Legionella pneumophila*, Survival, Freshwater, Stringent response, Transcriptome, Antibiotic resistance, *bdhA*

## Abstract

**Background:**

*Legionella pneumophila* (*Lp*) is a water-borne opportunistic pathogen. In water, *Lp* can survive for an extended period of time until it encounters a permissive host. Therefore, identifying genes that are required for survival in water may help develop strategies to prevent *Legionella* outbreaks.

**Results:**

We compared the global transcriptomic response of *Lp* grown in a rich medium to that of *Lp* exposed to an artificial freshwater medium (Fraquil) for 2, 6 and 24 hours. We uncovered successive changes in gene expression required for the successful adaptation to a nutrient-limited water environment. The repression of major pathways involved in cell division, transcription and translation, suggests that *Lp* enters a quiescent state in water. The induction of flagella associated genes (*flg*, *fli* and *mot*), enhanced-entry genes (*enh*) and some Icm/Dot effector genes suggests that *Lp* is primed to invade a suitable host in response to water exposure. Moreover, many genes involved in resistance to antibiotic and oxidative stress were induced, suggesting that *Lp* may be more tolerant to these stresses in water. Indeed, *Lp* exposed to water is more resistant to erythromycin, gentamycin and kanamycin than *Lp* cultured in rich medium. In addition, the *bdhA* gene, involved in the degradation pathway of the intracellular energy storage compound polyhydroxybutyrate, is also highly expressed in water. Further characterization show that expression of *bdhA* during short-term water exposure is dependent upon RpoS, which is required for the survival of *Lp* in water. Deletion of *bdhA* reduces the survival of *Lp* in water at 37 °C.

**Conclusions:**

The increase of antibiotic resistance and the importance of *bdhA* to the survival of *Lp* in water seem consistent with the observed induction of these genes when *Lp* is exposed to water. Other genes that are highly induced upon exposure to water could also be necessary for *Lp* to maintain viability in the water environment.

**Electronic supplementary material:**

The online version of this article (doi:10.1186/s12864-015-1869-6) contains supplementary material, which is available to authorized users.

## Background

*Legionella pneumophila* (*Lp*) is a Gram-negative bacterium that inhabits natural freshwater environments and man-made water systems. *Lp* has a broad host range and is able to replicate in different species of amoeba, ciliated protozoa and slime mold [1]. Importantly, it is also able to infect humans, resulting in a potentially fatal illness called Legionnaires’ disease (LD) [2]. Human infection occurs through inhalation of aerosols originating from *Legionella*-contaminated sources, such as cooling towers, air conditioning and heating systems, fountains and even showers [3]. Upon entry into alveolar macrophages, *Lp* prevents fusion with lysosomes, modulates host cell trafficking, forms a *Legionella*-containing vacuole and then starts intracellular multiplication [4]. In recent years, the occurrence rate of LD has been reported to be on the increase in many countries [5]. In the US, the incidence rate of LD increased by 192 % over the last decade [6]. In Europe, France, Italy and Spain consistently have the highest number of reported cases, with 7.06 to 11.7 cases per thousand in 2011 [7]. Most of these are sporadic cases, either community-acquired, nosocomial or travel-associated [8]. Nevertheless, outbreak of LD in which a large population gets exposed to contaminated aerosols from a point-source is of great concern. Investigations of previous outbreaks show that the dispersal distance of *Lp* from cooling towers and air scrubbers can be greater than 10 km [9, 10].

Many bacteria, such as *Bacillus megaterium*, *Salmonella enteritidis*, *Staphylococcus aureus* and *Vibrio cholera*, die steadily upon exposure to freshwater [11, 12]. Chandran et al. [13] showed that the colony-forming units (CFU) of *Escherichia coli* and *Vibrio parahaemolyticus* decreased by 7 logs after 27 days of exposure to lake water. In contrast, *Lp* is able to survive for a prolonged period of time in freshwater despite the lack of nutrients [14–17]. In an earlier study, *Lp* exposed to drinking water and creek water had a mere 2 log reduction in CFU counts and still maintained 3 × 10^6^ CFU/ml after an incubation time of 1.5 years [18]. The ability of *Lp* to survive in water for long periods is essential for the colonization of water systems, allowing it to persist until optimal conditions and permissive hosts for its growth are encountered. Survival in water is, therefore crucial for the transmission of *Lp* to the human host. In turn, it is important to understand the genetic factors of *Lp* that allow its prolonged survival in water. This knowledge may allow future development of strategies that prohibit the survival of *Lp* in water systems and eventually help control *Legionella* outbreaks.

*Lp* possesses approximately 3000 genes, in which 2434 genes are commonly found in all six strains [19]. As a human pathogen, the genes involved in virulence have been extensively studied [20–23]. Two major secretion systems, the Lsp type II secretion system (T2SS) and the Icm/Dot type IVB secretion system (T4BSS), translocate more than 300 effector proteins into the host cell and are critical for the virulence of *Lp* [24]. Moreover, many other genes act as virulence factors that directly contribute to host cell infection (e.g., *mip*, *enhABC*) or as virulence regulators (e.g., *rpoS*, *cpxR* and *letA*) [25–29]. Apart from the virulence genes, a recent study has showed that at least 597 genes are essential for optimal growth of *Lp* in rich medium [30]. However, the functions of many *Lp* genes still remains unknown. To date, only a few genes are known to be important for the survival of *Lp* in water. Söderberg et al. [17] studied the survival of *lspD*, *lspE*, *lspF* and *pilD* mutants in tap water and concluded that the T2SS is important for *Lp* to maintain survival at temperatures between 4 and 17 °C. Recently, our group has shown that the sigma factor RpoS and the stringent response (RelA and SpoT) are required for the survival of *Lp* in water [31].

Given the lack of knowledge and the associated risk to public health, it is necessary to identify more *Lp* genes that are required for survival in water. Bacteria typically respond to environmental changes through transcriptomic reorganization, where they express genes that are essential for coping with the new condition and repress genes that are no longer required, or that are detrimental [32, 33]. Comparison of transcriptional changes using microarrays can be used to identify candidate genes needed in a particular condition [34–36]. Recently, this technique has been used to identify a new gene, *iroT*, involved in ferrous ion transport based on the transcriptomic profile of *Lp* in an iron restricted condition [37].

In this study, we use a transcriptomic approach to identify genes that are potentially involved in the survival of *Lp* in water. Since bacteria tend to have immediate transcriptomic responses within the first few hours upon exposure to stressful conditions [38], the transcriptomic response of *Lp* to water was studied at an early time point (2 h), an intermediate time point (6 h) and a late time point (24 h). Genes involved in adaptation and regulatory functions are induced in water, while those involved in energy metabolism and translation are repressed. In particular, our analysis shows that *bdhA* is strongly expressed upon exposure to water, and the deletion of *bdhA* reduces the survival of *Lp* in water at 37 °C.

## Results

### Survival of *Lp* in Fraquil

**Fig. 1:**
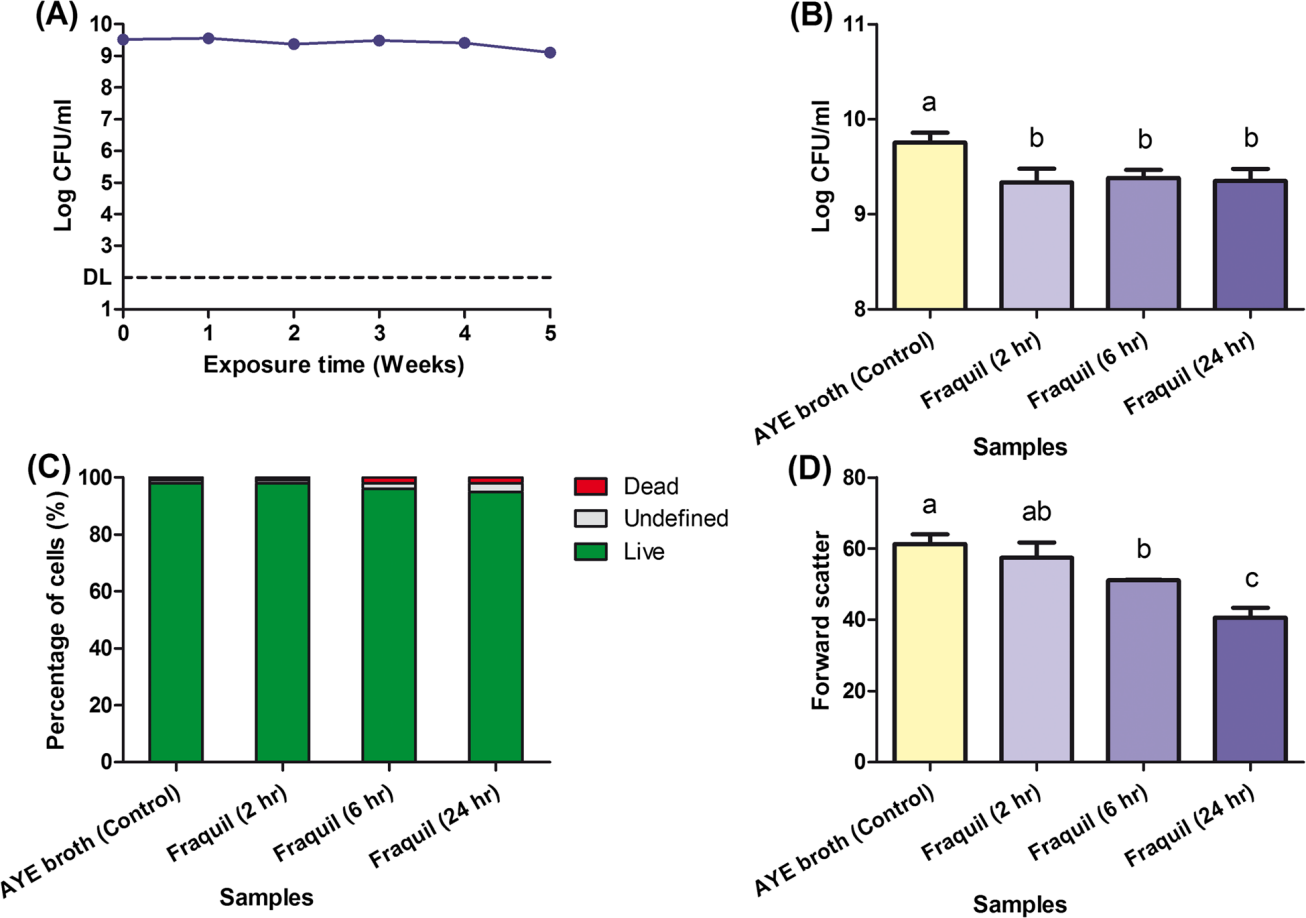
*Lp* survives well in water at 25 °C. **a** CFU counts of JR32 during five weeks of exposure to Fraquil. DL indicates the detection limit at 100 CFU/ml. **b** CFU counts of JR32 cultured in AYE broth or exposed to Fraquil. All samples were at an OD_600_ of 1.0. Data shows the mean +/− SD of three biological replicates. Different letters on the bars indicate significant differences between the samples according to Tukey's test (*p* < 0.05). **c** Percentage of live, undefined or dead JR32 cells cultured in AYE broth or exposed to Fraquil. Live/Dead staining was used with flow cytometry to determine the status of 5000 cells in each sample. **d** Forward scatter (FSC) of JR32 cultured in AYE broth or exposed to Fraquil. Each sample had three biological replicates and the mean FSC signal of 5000 cells in each replicate was detected by flow cytometry

In order to ensure reproducibility, an artificial freshwater medium was used to perform the transcriptomic analysis. The freshwater medium Fraquil was selected for this purpose since it mimics the composition of freshwater in North America [39]. The survival of the wild-type strain JR32 in Fraquil at 25 °C is shown in Fig. 1a. The population of *Lp* in Fraquil was stable for at least five weeks.

For the transcriptomic analysis, JR32 was first cultured in ACES-buffered yeast extract (AYE) broth to exponential phase in triplicate and 1 ml samples were harvested to serve as controls. Then, the cells were washed three times with Fraquil and re-suspended in Fraquil at a final OD_600_ of 1.0. The three re-suspensions were transferred to vessels of a BIOSTAT® Q Plus bioreactor to control temperature and dissolved oxygen. Samples were taken after 2, 6 and 24 h of exposure (treatment) and compared to controls grown in AYE broth. At each time point, we performed both CFU counts and Live/Dead staining. Although both AYE samples and Fraquil samples were at the same optical density, the CFU counts of JR32 exposed to Fraquil were consistently lower, albeit by a very small margin, than JR32 from AYE broth (Fig. 1b). There were no significant differences in CFU counts between the Fraquil samples at the different time points tested. Flow cytometry analysis of Live/Dead staining was used to evaluate the percentage of viable cells and showed that there were no differences between samples (Fig. 1c). Taking into account CFU counts and cell staining, *Lp* showed no significant survival defects within the first 24 h of exposure to Fraquil.

Based on the flow cytometry data, a steady reduction in the forward scatter (FSC) was observed in JR32 upon exposure to Fraquil (Fig. 1d). The lowest average FSC signal was observed in Fraquil samples after 24 h (the longest exposure time). Since the FSC signal is proportional to particle size [40], this result indicates that *Lp* undergoes a gradual reduction in cell size after exposure to water.

### Transcriptomic response of *Lp* exposed to water

In order to understand the genetic regulation of *Lp* during short-term exposure to water, we performed a transcriptomic analysis through DNA microarray hybridization. RNA was extracted from exponential phase (Control) and Fraquil-treated (Treatment) samples and the data of each treatment was compared with the control.

**Fig. 2:**
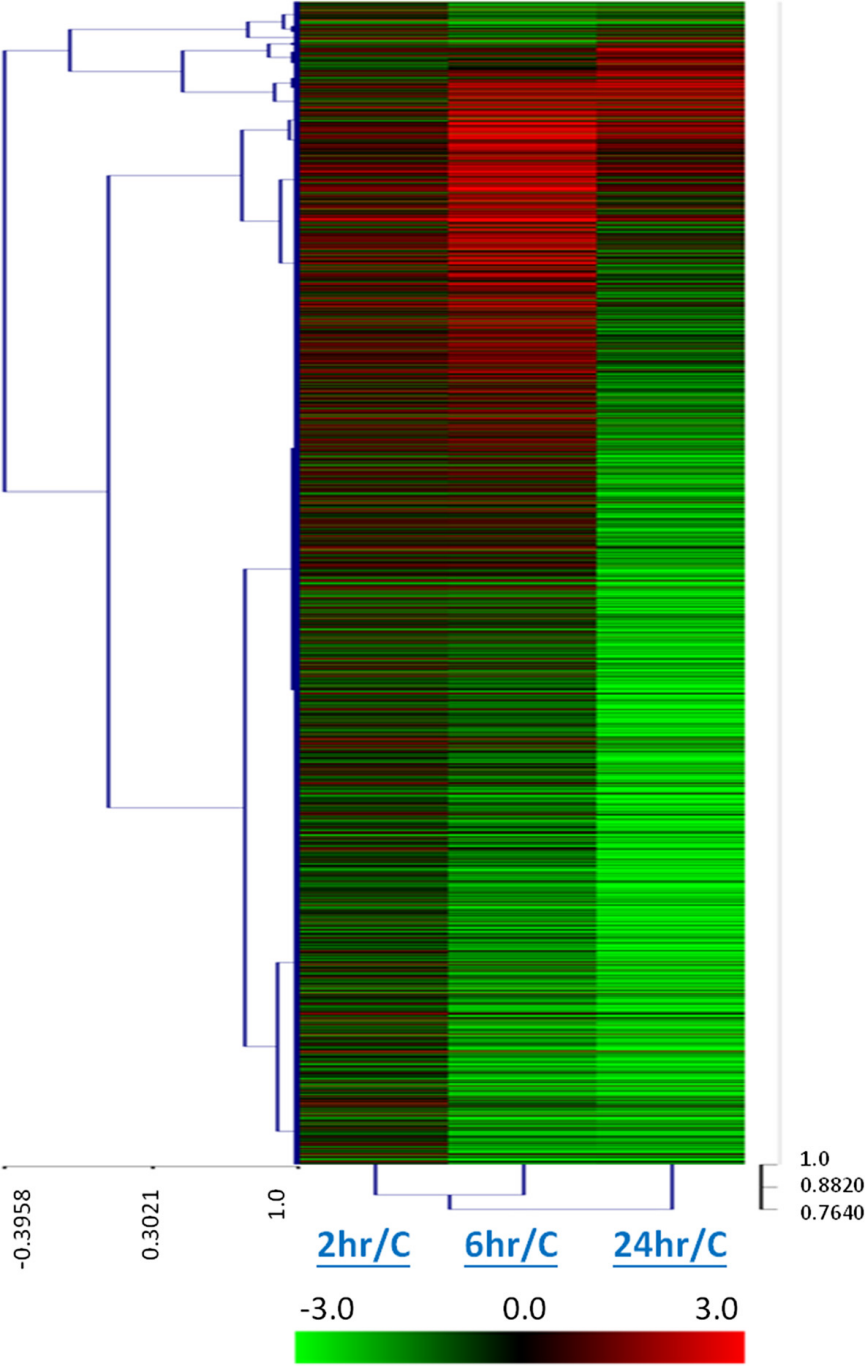
Progressive transcriptomic changes of *Lp* in water. The global gene expression of JR32 after 2, 6 and 24 h exposure to Fraquil in comparison with the control cultured in AYE broth is shown in the heat map. The hierarchical clustering shows the similarities between samples. The genes that were up-regulated are shown in red and those down-regulated are shown in green

**Fig. 3:**
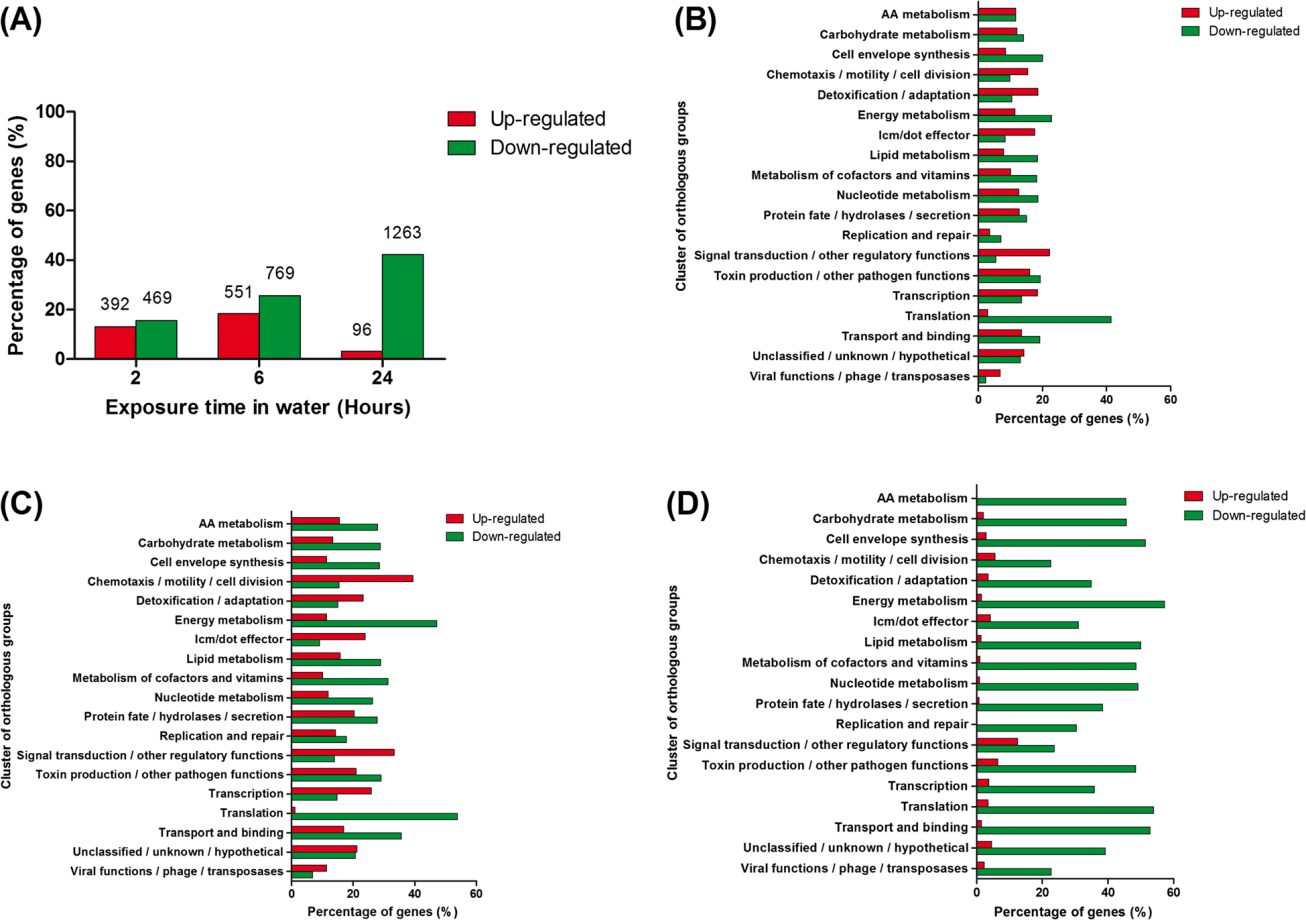
Many *Lp* genes are shut down after 24 h of exposure to water. **a** Percentage of genes significantly up-regulated or down-regulated after 2, 6 and 24 h of exposure to Fraquil. The numbers on the top of each bar represent the number of genes differently expressed (Log_2_ < −1 or >1, *p* < 0.05) over a total of 2994 annotated genes from the original genome annotation. Cluster of orthologous groups analysis of JR32 genes after (**b**) 2 h, (**c**) 6 h and (**d**) 24 h of exposure to Fraquil is shown. Up-regulated genes are shown in red and down-regulated genes are shown in green

Compared to JR32 growing in AYE broth, the expression of 2080 annotated genes and 201 predicted sRNA encoding sequences changed significantly (log_2_ ratio of Treatment/Control >1 or < −1, *p* < 0.05), in at least one time point (2, 6 or 24 h) after exposure to Fraquil (Additional file 1). A progressive transcriptomic change over time is clearly seen in the heat map showing the induction and repression of genes (Fig. 2). The percentages of significantly up- and down-regulated genes increased from 2 to 6 h of water exposure, while more genes were significantly down-regulated than up-regulated after 24 h (Fig. 3a). There were 13.1 % up-regulated genes and 15.7 % down-regulated genes after 2 h of water exposure, demonstrating the rapid transcriptional responses of *Lp* upon exposure to Fraquil.

Genes that were differentially expressed after water exposure were categorized into orthologous groups. After 2 and 6 h of water exposure, seven of the 19 orthologous groups had a higher percentage of up-regulated genes (Fig. 3b and c). These orthologous groups are “chemotaxis/motility/cell division”, “detoxification/adaptation”, “icm/dot effector”, “signal transduction/other regulatory functions”, “transcription”, “unclassified/unknown/hypothetical” and “viral functions/phage/transposases”. In contrast, all 19 orthologous groups showed major down-regulation of genes after 24 h of water exposure (Fig. 3d). At each time point, “energy metabolism”, “translation” and “transport and binding” remained the top three orthologous groups with the highest percentage of down-regulated genes. Examples of genes in these orthologous groups are listed in Table 1 and 2 and will be discussed later.

**Table 1:** Selected genes significantly down-regulated in water

			Log_2_ ratio^a^		
Functional class and protein	Locus tag	Gene	2 hr/C	6 hr/C	24 hr/C
Cell division					
Cell division protein	*lpg2610*	*ftsA*		−1.12	−2.93
Cell division protein	*lpg2611*	*ftsQ*	−1.18	−1.28	−4.43
Cell division protein	*lpg2615*	*ftsW*		−2.34	−5.59
Energy metabolism					
ATP synthase F_0_, A subunit	*lpg2988*	*atpB*	−1.38	−2.58	−3.49
ATP synthase F_0_, C subunit	*lpg2987*	*atpE*		−1.27	−2.58
ATP synthase F_0_, I subunit	*lpg2989*	*atpI*		−1.13	−2.51
ATP synthase F_1_, alpha subunit	*lpg2984*			−1.77	−1.71
ATP synthase F_1_, beta subunit	*lpg2982*	*atpD*	−1.19	−2.53	−2.44
ATP synthase F_1_, epsilon subunit	*lpg2981*	*atpC*		−1.67	−4.33
ATP synthase F_1_, gamma subunit	*lpg2983*	*atpG*	−1.36	−2.16	−2.60
ATP synthase F_1_,delta subunit	*lpg2985*	*atpH*		−1.89	−2.15
Cytochrome c oxidase, subunit I	*lpg2896*			−1.71	−3.10
Cytochrome c oxidase, subunit II	*lpg2897*			−2.38	−3.10
Cytochrome c	*lpg2898*			−1.16	−1.64
NADH dehydrogenase I, A subunit	*lpg2789*	*nuoA*	−1.56	−3.42	−6.44
NADH dehydrogenase I, B subunit	*lpg2788*	*nuoB2*	−4.60	−6.26	−8.44
NADH dehydrogenase I, C subunit	*lpg2787*	*nuoC*	−1.16	−3.34	−4.65
NADH dehydrogenase I, H subunit	*lpg2782*	*nuoH*	−1.72	−3.71	−6.66
NADH dehydrogenase I, I subunit	*lpg2781*	*nuoI*	−1.00	−2.82	−4.16
Succinate dehydrogenase	*lpg0528*	*sdhC*			−2.94
Succinate dehydrogenase	*lpg0530*	*sdhA*			−2.48
Succinate dehydrogenase	*lpg0531*	*sdhB*	−1.01		−2.93
Ubiquinol-cytochrome c reductase	*lpg2704*	*petB*		−1.66	−5.08
Ubiquinol-cytochrome c reductase	*lpg2705*	*petA*		−1.46	−2.54
Signal transduction/other regulatory functions					
Response regulator	*lpg1912*	*letS*		−1.15	
Response regulator	*lpg2646*	*letA*	−4.02	−3.57	−5.46
Toxin production/other pathogen functions					
Macrophage infectivity potentiator	*lpg0791*	*mip*	−1.22		−2.04
Transcription					
DNA-directed RNA polymerase alpha subunit	*lpg0354*	*rpoA*	−2.70	−3.95	
DNA-directed RNA polymerase beta subunit	*lpg0322*	*rpoB*	−1.74	−2.63	
DNA-directed RNA polymerase beta' subunit	*lpg0323*	*rpoC*			−2.83
RNA polymerase sigma-32 factor	*lpg2667*	*rpoH*			−2.09
Translation					
30S ribosomal protein S13	*lpg0351*	*rpsM*	−2.57	−4.22	−5.00
30S ribosomal protein S20	*lpg2636*	*rpsT*	−3.86	−4.43	−6.92
30S ribosomal protein S6	*lpg1592*	*rpsF*	−2.99	−4.18	−4.84
30S ribosomal protein S7	*lpg0325*	*rpS7*	−2.43	−3.36	−3.89
50S ribosomal protein L15	*lpg0348*	*rplO*	−1.76	−3.37	−3.21
50S ribosomal protein L16	*lpg0336*	*rplP*	−2.49	−3.73	−3.21
50S ribosomal protein L28	*lpg0479*	*rpmB*	−2.00	−2.89	−3.40
50S ribosomal protein L6	*lpg0344*	*rplF*	−2.49	−3.81	−3.47
Translation elongation factor G	*lpg0326*	*fusA*	−2.45	−2.59	
Translation elongation factor Ts	*lpg1713*	*tsf*		−1.40	−1.20
Translation initiation factor IF-1	*lpg1770*	*infA*	−2.47	−3.69	−5.00
Translation initiation factor IF-3	*lpg2713*	*infC*	−3.20	−3.29	
tRNA-Gly	*lpg2292*		−3.05	−3.10	−3.76
tRNA-Met	*lpg0797*		−2.99	−3.68	−5.53
tRNA-Phe	*lpg1929*		−3.29	−3.63	−4.44
tRNA-Pro	*lpg1866*		−3.54	−4.49	−5.83
Transport and binding					
Amino acid antiporter	*lpg1658*		−2.19	−3.66	−5.86
Amino acid antiporter	*lpg1691*		−2.89	−3.61	−4.31
Amino acid antiporter	*lpg1917*		−2.51	−4.11	−4.80
Amino acid permeases	*lpg0970*		−1.91	−3.42	−5.45
Amino acid permease family protein	*lpg0228*		−2.77	−5.41	−6.13
Amino acid transporter	*lpg0049*				−3.27
Ferrous iron transporter	*lpg2658*	*feoA*	−2.56	−2.80	−4.47
Ferrous iron transporter	*lpg2657*	*feoB*		−3.10	−5.23
DotA	*lpg2686*	*dotA*			−1.35
DotC	*lpg2675*	*dotC*	−1.40	−2.29	−4.72
DotD	*lpg2674*	*dotD*		−1.46	−3.67
DotK	*lpg0447*	*lphA*	−2.59	−1.69	−5.16
IcmB (DotO)	*lpg0456*	*icmB*			−3.00
IcmC (DotE)	*lpg0453*	*icmC*		1.27	−1.11
IcmF	*lpg0458*	*icmF*			−1.13
IcmH (DotU)	*lpg0459*	*icmH*	−2.95	−2.42	−5.33
IcmJ (DotN)	*lpg0455*	*icmJ*			−1.69
IcmK (DotH)	*lpg0450*	*icmK*		−2.10	−4.11
IcmL (DotI)	*lpg0449*	*icmL*		−1.93	−5.42
IcmL homolog	*lpg0708*				−2.65
IcmL homolog	*lpg0383*				−2.81
IcmM (DotJ)	*lpg0448*	*icmM*			−2.57
IcmO (DotL)	*lpg0446*	*icmO*		2.07	−1.50
IcmQ	*lpg0444*	*icmQ*			−1.36
IcmR	*lpg0443*	*icmR*			−2.16
IcmS	*lpg0442*	*icmS*			−2.43
IcmT	*lpg0441*	*icmT*	1.39	1.79	−1.09
IcmV	*lpg2687*	*icmV*	1.00		−1.89
IcmW	*lpg2688*	*icmW*			−3.09
IcmX (IcmY)	*lpg2689*	*icmX*			−1.41
Type IV pilus biogenesis protein	*lpg0927*	*pilM*			−2.04
Type IV pilus biogenesis protein	*lpg0928*	*pilN*	−1.36		−2.74
Type IV pilus biogenesis protein	*lpg0930*	*pilP*			−3.66

^a^Only values that were significantly different than the control are shown

**Table 2:** Selected genes significantly up-regulated in water

			Log_2_ ratio^a^		
Functional class and protein	Locus tag	Gene	2 hr/C	6 hr/C	24 hr/C
Chemotaxis/motility					
Flagellar assembly protein	*lpg1790*	*fliO*		5.35	
Flagellar basal body rod protein	*lpg1221*	*flgG*	2.38	3.01	
Flagellar biosynthesis sigma factor	*lpg1782*	*fliA*		3.34	1.53
Flagellar biosynthetic protein	*lpg1788*	*fliQ*		2.75	
Flagellar biosynthetic regulator	*lpg1784*	*flhF*		3.46	
Flagellar hook protein	*lpg1219*	*flgE*		4.17	3.79
Flagellar L-ring protein	*lpg1222*	*flgH*		2.13	
Flagellar motor protein	*lpg2318*	*motA*		1.63	
Flagellar motor protein	*lpg1780*	*motB*		2.72	
Flagellar motor protein	*lpg1781*	*motC*		4.02	
Flagellar motor switch protein	*lpg1792*	*fliM*		3.48	1.44
Flagellar P-ring protein	*lpg1223*	*flgI*		2.99	
Flagellin	*lpg1340*	*fliC*	1.22	1.71	
Detoxification/adaptation					
Alkylhydroperoxidase	*Lpg2349*	*ahpD*	2.63	2.25	
Alkylhydroperoxide reductase	*lpg2350*	*ahpC*	1.74	1.64	
Alkylhydrogen peroxide reductase	*lpg2965*	*ahpC*	1.62	1.85	
Heat shock protein	*lpg2024*	*dnaJ*	1.56	1.96	
Heat shock protein	*lpg2817*	*yrfI*	1.62	2.77	
Aminoglycoside 6-adenylyltransferase	*lpg2151*		1.80	3.01	2.11
Spectinomycin phosphotransferase	*lpg1492*		1.74	1.73	
Stress-induced protein	*lpg2011*		3.81	5.97	
Superoxide dismutase	*lpg2348*	*sodC*	2.45	2.88	
Universal stress protein A	*lpg0935*			1.59	
Icm/dot effector					
Coiled-coil-containing protein	*lpg1488*	*legC5*	1.75	1.05	
F-box protein	*lpg2144*	*legAU13*	1.07	1.74	2.11
Hypothetical	*lpg0096*	*ceg4*	2.69	2.16	
Hypothetical	*lpg2591*	*ceg33*	3.35	4.83	
Hypothetical	*lpg1290*	*lem8*	1.59	2.48	2.20
Hypothetical	*lpg1496*	*lem10*	1.51	2.79	3.28
Protein SdhA	*lpg0376*	*sdhA*	1.31	1.60	
Protein SidA	*lpg0621*	*sidA*		2.47	
Sid related protein-like	*lpg2157*	*sdeA*		2.33	1.60
UBOX-containing protein	*lpg2830*	*legU2*	1.35	2.34	
UVB-resistance protein	*lpg1976*	*legG1*	1.66	1.65	
Lipid metabolism					
3-hydroxybutyrate dehydrogenase	*lpg2316*	*bdhA*		2.40	1.31
Other functions					
6S RNA	*ssrS*			3.65	3.55
RsmY	*rsmY*				2.57
RsmZ	*rsmZ*			1.58	1.80
Signal transduction/other regulatory functions					
Response regulator TutC	*lpg2146*			2.39	1.10
Sensor histidine kinase	*lpg0230*	*pleD*		2.95	2.27
Sensor protein LuxN	*lpg2734*		1.23	1.89	
Sensory box protein, GGDEF/EAL domain	*lpg0029*	*rre41*	1.33	1.07	
Sensory box protein, GGDEF/EAL domain	*lpg1025*	*yegE*	1.37	4.85	2.16
Serine/threonine-protein kinase	*lpg0208*	*pkn5*		1.25	
Sigma 54 modulation protein YhbH	*lpg1206*			3.95	4.04
Signal transduction protein	*lpg0156*		3.11	5.19	
Toxin production / other pathogen functions					
Enhanced entry protein EnhA	*lpg1336*	*enhA*	1.10	2.06	
Enhanced entry protein EnhA	*lpg1386*			1.28	1.31
Enhanced entry protein EnhA	*lpg2641*	*enhA*	2.57	3.75	
Enhanced entry protein EnhA	*lpg0910*	*enhA*		1.72	3.18
Enhanced entry protein EnhB	*lpg2640*	*enhB*		2.40	
Enhanced entry protein EnhC	*lpg1172*			2.58	5.87
Enhanced entry protein EnhC	*lpg1356*			2.70	2.88
RtxA	*lpg0645*		1.57	3.04	
Transcription					
DNA binding protein	*lpg2441*		1.79		
RNA polymerase sigma-54 factor	*lpg0477*			1.44	
Transcription repair coupling factor	*lpg0954*	*mfd*	2.04	2.14	
Transcriptional regulator	*lpg0586*		1.24	3.75	4.48
Transcriptional regulator, ArsR family	*lpg2723*		3.63	1.18	
Transcriptional regulator, AsnC family	*lpg1486*			2.33	
Transcriptional regulator, DeoR family	*lpg2167*			1.76	
Transcriptional regulator, LuxR family	*lpg2524*			1.52	1.73
Transcriptional regulator, LysR family	*lpg2138*		3.58	4.21	
Transcriptional regulator, MarR family	*lpg2140*		1.80	1.90	
Transport and binding					
IcmC (DotE)	*lpg0453*	*icmC*		1.27	−1.11
IcmC homolog (DotV)	*lpg0472*		2.06	2.69	
IcmD (DotP)	*lpg0454*	*icmD*	1.25	1.77	
IcmO (DotL)	*lpg0446*	*icmO*		2.07	−1.50
IcmP (DotM)	*lpg0445*	*icmP*	2.15	3.54	
IcmT	*lpg0441*	*icmT*	1.39	1.79	−1.09
IcmV	*lpg2687*	*icmV*	1.00		−1.89
Erythromycin resistance protein/ABC transporter	*lpg1616*	*uup*	1.86	1.61	
Multidrug resistance protein/efflux pump	*lpg0257*		2.67	3.09	
Multidrug resistance protein/efflux pump	*lpg0429*	*oprM*	1.12	1.65	
Multidrug resistance protein/efflux pump	*lpg2189*	*ygjT*	1.76	2.28	−1.85
Viral functions/phage/transposases					
Prophage regulatory protein	*lpg2563*		1.22		
Transposase	*lpg2120*			2.01	
Transposase	*lpg2363*		1.65		

^a^Only values that were significantly different than the control are shown

### Validation of microarray results by RT-qPCR

To validate the results of the microarray analysis, the expression profiles of ten different genes were confirmed by reverse transcription quantitative PCR (RT-qPCR) using 16 s rRNA as an internal control [41, 42]. These ten genes were selected from eight different orthologous groups.

**Fig. 4:**
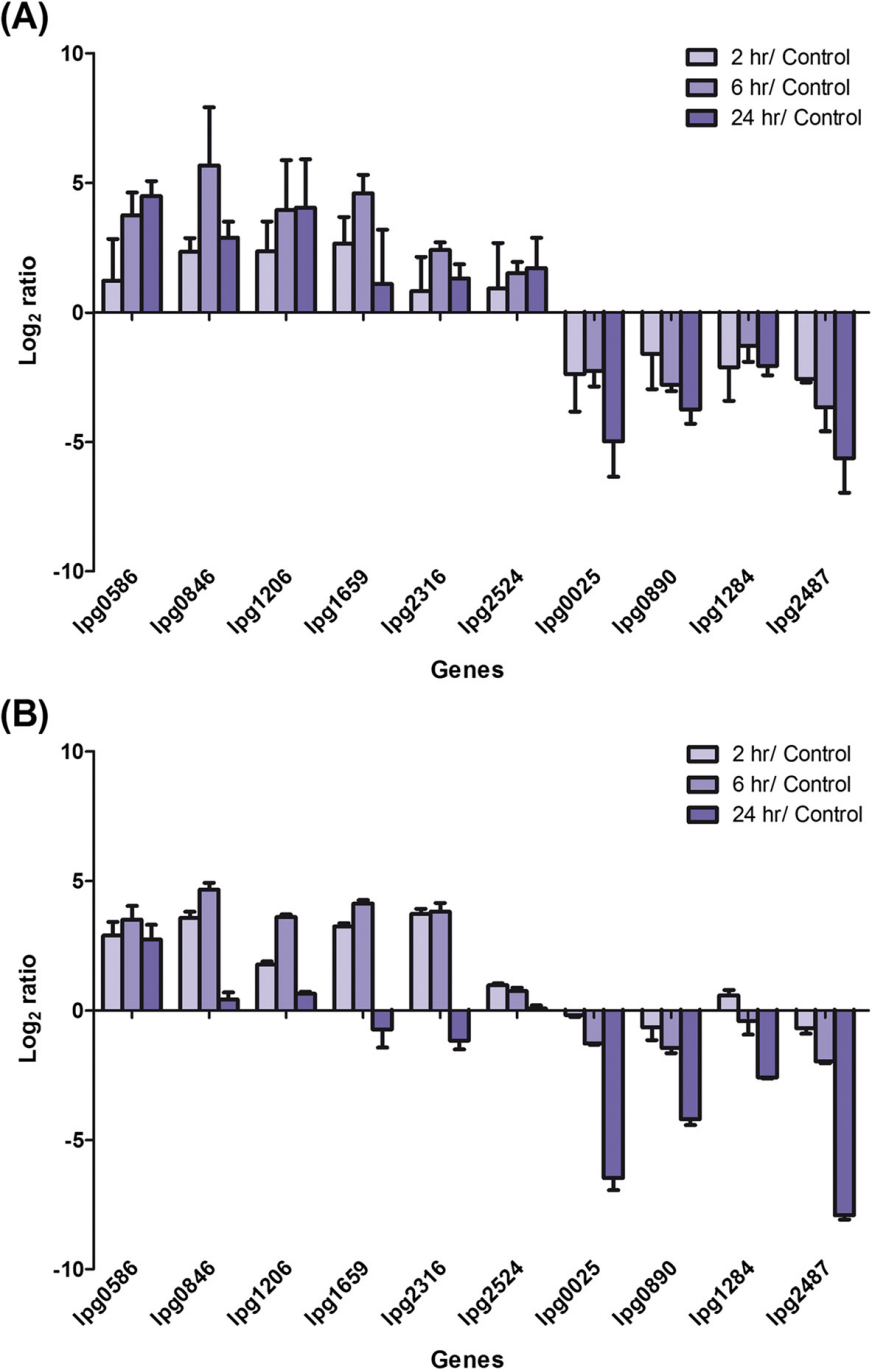
Microarray data is validated by RT-qPCR. Transcriptomic changes of ten selected JR32 genes upon water exposure shown by (**a**) microarray analysis and (**b**) RT-qPCR. Results are shown as the log_2_ ratio between the samples exposed to Fraquil for 2, 6 or 24 h and the control cultured in AYE broth

According to the microarray data, six of the selected genes (*lpg0586*, *lpg0846*, *lpg1206*, *lpg1659*, *lpg2316* (*bdhA*) and *lpg2524*) were significantly up-regulated in water at two or all three time points, while the remaining four genes (*lpg0025*, *lpg0890*, *lpg1284* and *lpg2487*) were significantly down-regulated (Fig. 4a). The RT-qPCR results for these 10 genes are shown in Fig. 4b. Despite some differences, the general expression profiles were conserved between the microarray and RT-qPCR data, which validates the former.

### Antibiotic resistance of *Lp* subsequent to water exposure

Many genes involved in detoxification and adaptation were induced in water, including some associated with antibiotic resistance, such as an aminoglycoside 6-adenylyltransferase, a spectinomycin phosphotransferase, an erythromycin transporter and several efflux pumps (Table 2). The induction of these genes may increase the resistance of water-exposed *Lp* to antibiotics. To investigate this hypothesis, we compared the antibiotic resistance of *Lp* cultured in AYE broth to exponential phase and those exposed to Fraquil for 24 h. In both cases, we compared the CFU counts with and without the addition of a β-lactam (ampicillin), two aminoglycosides (gentamycin and kanamycin) and a macrolide (erythromycin). In order to determine the impact of temperature on antibiotic resistance, *Lp* was grown in AYE and exposed to Fraquil at both 25 °C and 37 °C prior to testing resistance.

**Fig. 5:**
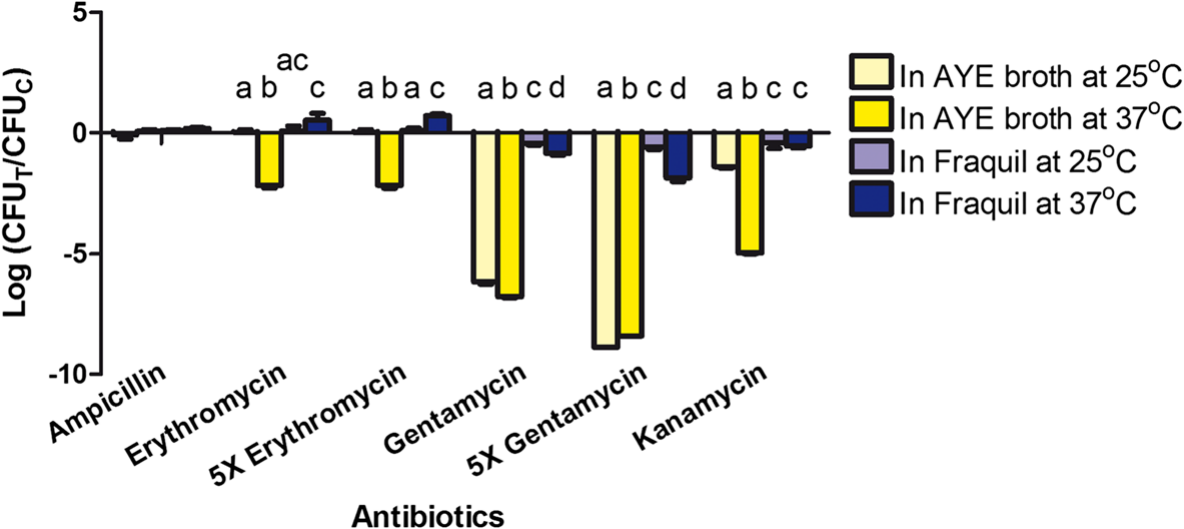
*Lp* exposed to water is more resistant to antibiotics. CFU changes of *Lp* cultured in AYE broth or exposed to Fraquil at 25 or 37 °C after antibiotic treatments. The antibiotics used include ampicillin, erythromycin, gentamycin and kanamycin, all at 100 μg/ml. 5X indicates a five times concentration of erythromycin and gentamycin (500 μg/ml) were used as well. The data are expressed as the log transformation of CFUs in treated wells (CFU_T_) divided by the CFUs in the control wells without antibiotics (CFU_C_). A negative value indicates a CFU reduction upon treatment. Data shows the mean +/− SD of three biological replicates. Different letters on the bars indicate significant differences between different samples in a particular treatment according to Tukey's test (*p* < 0.05). CFU changes between different treatments were not compared

There were no significant differences in the CFU counts between AYE-grown and Fraquil-exposed *Lp* treated with ampicillin (Fig. 5). *Lp* grown in AYE at 37 °C was more susceptible to erythromycin than *Lp* grown in AYE at 25 °C or *Lp* exposed to Fraquil at either temperature. *Lp* exposed to Fraquil was significantly more resistant to gentamycin than *Lp* grown in AYE broth, regardless of the temperature. Moreover, exposure to 37 °C seems to slightly increase susceptibility to gentamycin. *Lp* was more resistant to kanamycin after exposure to Fraquil than when grown in AYE, and this difference was more pronounced at 37 °C. Overall, *Lp* is more resistant to aminoglycosides after exposure to Fraquil, which is consistent with our prediction based on the transcriptomic data. Incubation temperature also seems to affect antibiotic resistance, especially against erythromycin.

### Importance of *bdhA* for the survival of *Lp* in water

Genes that are significantly induced upon exposure to Fraquil may be important for *Lp* to survive in water. Upon this premise, the highly up-regulated *bdhA* gene was selected for further characterization. A deletion mutant of *bdhA* (Δ*bdhA*) and its complement (SPF236) were constructed. For the complementation, *bdhA* was cloned downstream of the *Ptac* promoter on pMMB207c. The wild-type strain KS79, Δ*bdhA*, SPF236 without isopropyl-β-D-thiogalactopyranoside (IPTG) (not induced) and SPF236 with 1 mM IPTG (induced) were exposed to Fraquil at 25 and 37 °C. CFU counts were monitored weekly and their survival was also assessed by Live/Dead staining after 19 weeks of exposure, when the CFUs of all strains at 37 °C dropped below the detection limit.

**Fig. 6:**
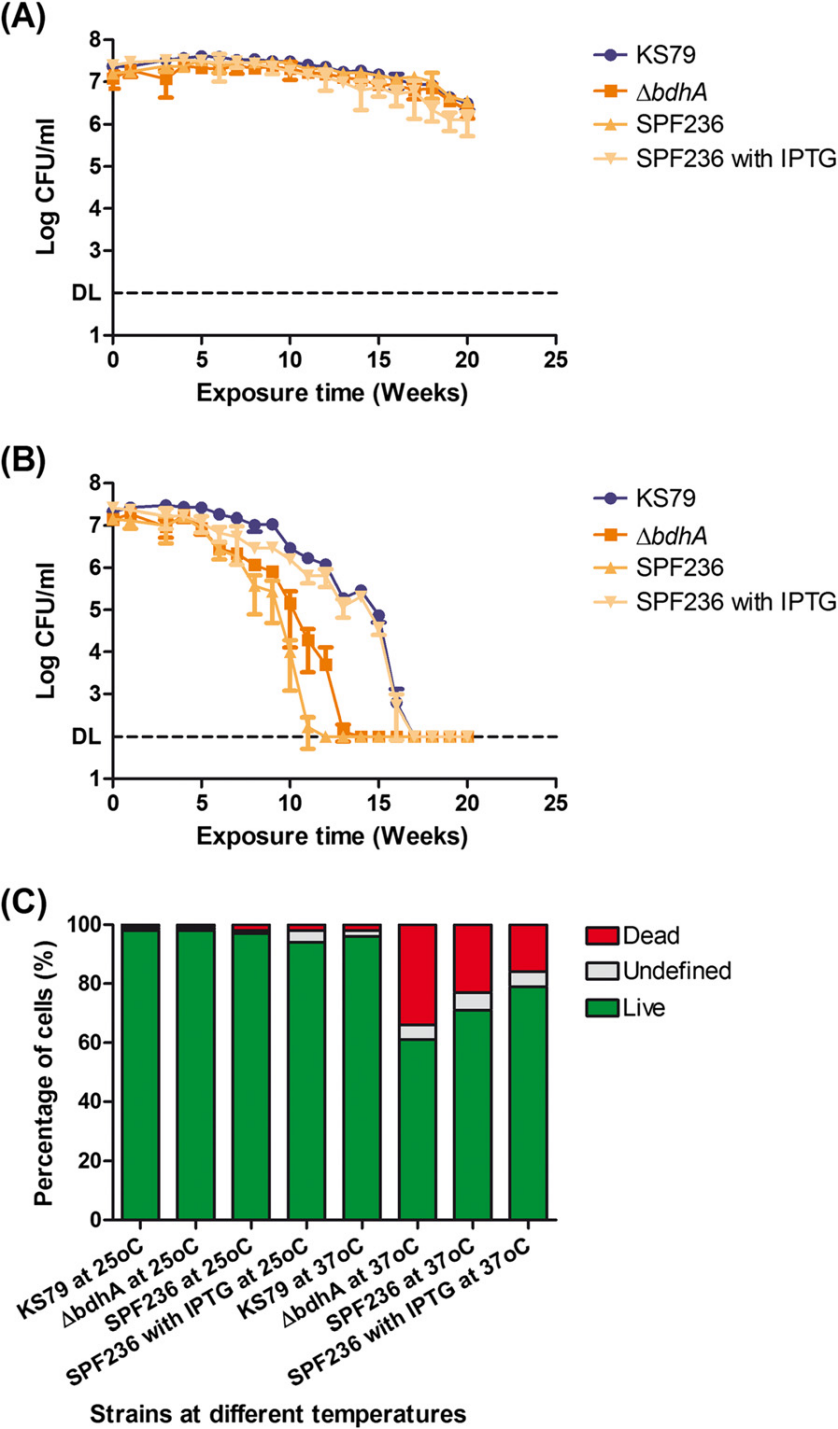
*bdhA* is important for long-term survival of *Lp* in water. Weekly CFU counts of the wild-type KS79, Δ*bdhA* and its complement SPF236 with and without IPTG exposed to Fraquil at (**a**) 25 °C and (**b**) 37 °C. 1 mM IPTG was used to induce *bdhA* on the plasmid of the complemented strain. Data show the mean +/− SD of three biological replicates. DL: Detection limit. **c** Percentage of live, undefined or dead cells of KS79, Δ*bdhA* and its complement SPF236 with and without IPTG. Live/Dead staining analysed by flow cytometry was performed after the strains were exposed to Fraquil for 19 weeks at 25 and 37 °C

No survival defects were observed in the mutant strain compared to the wild-type or the complements in Fraquil at 25 °C (Fig. 6a). However, a minor reduction (1 log) in the CFU counts was observed in all strains after 20 weeks of water exposure. An overall faster drop in CFUs was observed in all strains at 37 °C, compared to 25 °C (Fig. 6b). The *bdhA* mutant strain showed a more rapid reduction in CFUs than the wild-type strain. This phenotype was complemented by expression of *bdhA* from the *Ptac* promoter in the presence of IPTG (SPF236 with IPTG). The CFU count of SPF236 without IPTG was similar to the *bdhA* mutant strain. Therefore, *bdhA* seems to be required for the survival of *Lp* in water at 37 °C.

Since the CFU counts of all strains exposed to 37 °C decreased below the detection limit after 19 weeks of water exposure, we sought to determine if the cells entered a viable but non-culturable (VBNC) state at this time point [43]. To this end, we used a Live/Dead staining procedure together with flow cytometry. At 25 °C, no significant differences in the percentage of viable cells were observed between strains (Fig. 6c), which is consistent with the CFU counts. On the other hand, the wild-type strain showed a small fraction of dead cells at 37 °C, which is not significant compared to 25 °C. In comparison, only 61 % cells of the *bdhA* mutant strain were stained as viable. The complemented strain exposed to water in the presence of IPTG had a higher percentage of viable cells but was still lower than that of the wild-type strain. Taken together with the CFU counts, our results show that *Lp* enters a VBNC state after 19 weeks of exposure to Fraquil at 37 °C.

### Regulation of *bdhA* by RpoS

Hovel-Miner et al. [44] showed that the expression of *bdhA* in a rich medium is regulated by RpoS when *Lp* reaches the post-exponential phase. Moreover, transcriptomic analysis of the *rpoS* mutant in water suggests that the expression of *bdhA* is RpoS-dependant [31]. Therefore, we investigated this possibility of RpoS regulation of *bdhA* by using a green fluorescent protein (GFP) reporter assay [45]. Briefly, a plasmid carrying the *PbdhA*-GFP transcriptional fusion was constructed (pSF53) and transformed into JR32 and the *rpoS* mutant (JR32 pSF53 and *rpoS* pSF53). JR32 harbouring the pXDC31 plasmid, which expresses GFP from the *Ptac* promoter in the presence of IPTG, was used as positive control. JR32 and *rpoS* mutant strains with a plasmid containing no promoter upstream of the GFP encoding sequence (JR32 pSF78, *rpoS* pSF78) served as negative controls. Each strain was exposed to AYE broth or Fraquil for 24 h and the level of GFP in terms of green fluorescence signal was measured by flow cytometry.

**Fig. 7:**
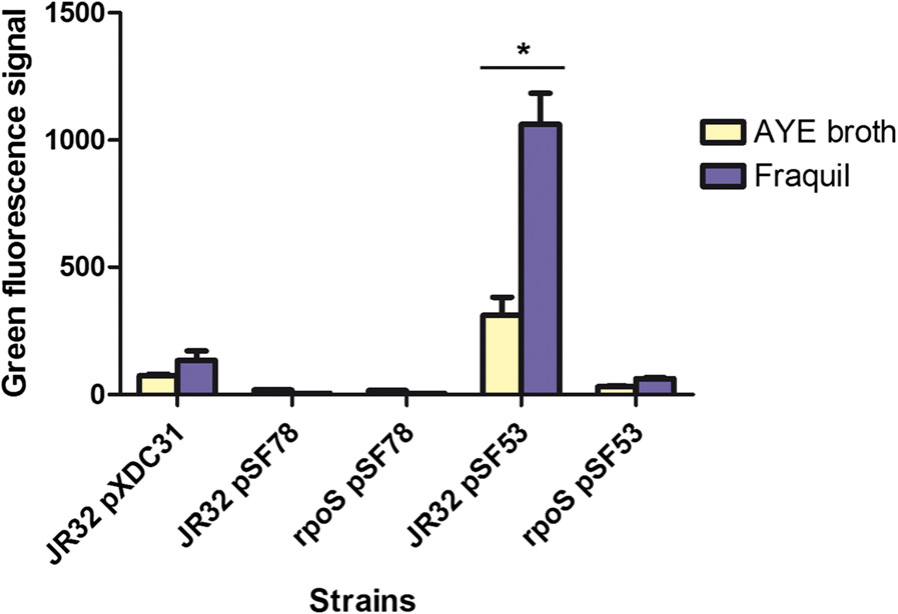
*bdhA* is highly induced in water and regulated by *rpoS*. Green fluorescence signal of five different strains after 24 h of exposure to AYE broth or Fraquil. JR32 pSF78, *rpoS* pSF78, JR32 pSF53 and *rpoS* pSF53 represent SPF265, SPF221, SPF266 and SPF211, respectively. JR32 pXDC31 was induced with 1 mM IPTG. Each sample had three biological replicates and the mean green fluorescence signal of 5000 cells in each replicate was detected by flow cytometry

A significantly higher level of GFP was found to be expressed in KS79 pSF53 exposed to Fraquil than that exposed to AYE broth, suggesting that the promoter of *bdhA* was more highly induced after 24 h in water (Fig. 7). This induction is consistent with the up-regulation of *bdhA (lpg2316)* observed in JR32 exposed to water for the same period of time during the transcriptomic analysis (Table 2). GFP expression in *rpoS* pSF53 was higher than in the negative controls under both conditions, but it was significantly lower than that of KS79 pSF53. In addition, no significant differences were found between *rpoS* pSF53 in AYE broth compared to Fraquil. These findings confirm that expression of *bdhA* in *Lp* is positively regulated by RpoS.

## Discussion

As a bacterium that lives in aqueous environments, *Lp* is frequently exposed to various stresses, such as fluctuations in temperature, pH and levels trace metals, as well as nutrient limitation [46]. Nutrient limitation, in particular, is a major concern as the nutrient levels in freshwater rarely fits the growth requirement of *Lp*, which is an auxotroph for at least eight amino acids [1, 47]. In the absence of essential nutrients or suitable hosts, *Lp* cannot grow, but can still persist in water for over one year [18]. Previous work has shown that the T2SS is important for the survival of *Lp* in water at low temperature [17]. However, no studies have investigated the global transcriptomic response of *Lp* to freshwater. In response to this knowledge gap, we performed a transcriptomic analysis to study the changes in gene expression of *Lp* exposed to Fraquil, an artificial freshwater medium composed of trace amounts of salts and metals, compared to *Lp* grown in rich medium (AYE broth). Since *Lp* in stationary phase culture is under nutrient limitation and stresses, using it as the control may hinder the opportunity to identify important genes for its survival in water. Therefore, *Lp* in the exponential phase of growth was used as the control.

In Fraquil, *Lp* maintained a stable population for at least 35 days and did not show any growth (Fig. 1a). Nevertheless, a significant reduction in cell size was found within 24 h of water exposure (Fig. 1d), which might be an adaptative response of *Lp* to nutrient limitation. In fact, the reduction of cell size is commonly observed in bacteria experiencing environmental stresses, such as nutrient limitation, suboptimal pH and low temperatures [48–52]. For example, the cell length of *V. parahaemolyticus* under starvation decreased drastically from 3.4 to 2.0 μm within 24 h [53]. Such a morphological change is believed to be a strategy to minimize the metabolic requirements for cell maintenance [54]. The mature intracellular form (MIF) of *Lp*, produced after passage through HeLa cells and *Tetrahymena tropicalis*, also shows a reduction in cell size, suggesting that this morphological change may be a response of *Lp* to nutrient limitation [55, 56].

The transcriptomic study of *Lp* revealed significant changes in gene expression following exposure to Fraquil. Since Fraquil does not contain any carbon sources that *Lp* can use, it is unable to grow in this medium (Fig. 1a). Consistent with this observation, expression of several amino acid transporters was induced in water (Table 1). Therefore, the reduced expression of three cell division proteins, such as *ftsA*, *ftsQ* and *ftsW*, was expected (Table 1). Moreover, bacteria tend to shut down major metabolic pathways when under starvation [57, 58]. Following exposure to water, genes involved in the electron transport chain (NADH dehydrogenase, succinate dehydrogenase, and cytochromes) and eight genes encoding subunits of the ATP synthase were significantly repressed (Table 1). This suggests a lower level of energy metabolism and thus, reduced metabolic activity in *Lp*, as the down-regulation of ATP synthase is usually found in dormant bacteria with a lower cellular ATP level [59]. Furthermore, compared to the control growing in AYE broth, the total RNA extracted from *Lp* exposed to water dropped markedly after 2 h and reduced to only one tenth of the control after 24 h (data not shown). This finding is consistent with the down-regulation of *rpoA*, *rpoB* and *rpoC*, which encode subunits of RNA polymerase, potentially resulting in less RNA polymerase available to bind to DNA and initiate transcription (Table 1).

In addition, most of the genes encoding 30S ribosomal proteins, 50S ribosomal proteins, translation initiation factors, translation elongation factors as well as tRNAs were down-regulated in water in two or all three time points tested (Table 1). In total, the expression of eight *rpm* genes, 18 *rps* genes and 19 *rpl* genes was significantly reduced (only some of these genes are shown in Table 1). In addition, *lpg1206* was strongly induced in water. This gene encodes a homolog of YhbH, which is a short hibernation promoting factor (HPF) with a highly conserved function in γ-Proteobacteria [60]. Short HPF stabilizes the dimerization of two 70S ribosomes into a translationally inactive 100S ribosome by a ribosome modulation factor, resulting in ribosome hibernation [61]. Ribosome hibernation is commonly found in bacteria experiencing nutrient limitation and is rapidly reversible when nutrients become available once again [62, 63]. In *E. coli*, a mutant unable to form 100S ribosomes survived poorly in the post-exponential phase [64]. Therefore, it is possible that the up-regulation of *lpg1206* in *Lp* may allow the formation of 100S ribosomes to maintain long-term survival in water, and the ability to resume growth or intracellular multiplication.

Our findings suggest that the translational machinery of *Lp* is shut down following exposure to water, which is a typical characteristic of the bacterial stringent response [65]. The stringent response is the reallocation of cellular resources in bacteria under nutrient limitation, by prohibiting the synthesis of DNA, stable RNAs, ribosomal proteins and membrane components, and activating the synthesis of factors involved in amino acid synthesis, glycolysis and stress resistance [65]. The massive transcriptomic changes during the stringent response are mediated by the synthesis and accumulation of guanosine tetraphosphate (ppGpp) and guanosine pentaphosphate (pppGpp), which are called (p)ppGpp collectively [65]. The stringent response in *Lp* has been studied in detail. Under amino acid limitation, the binding of uncharged tRNAs to the A site in ribosomes triggers the ribosomal-associated RelA protein to synthesize (p)ppGpp [29, 66, 67]. SpoT, the second stringent response protein, can also synthesize (p)ppGpp in response to other signals, such as perturbation of fatty acid synthesis [67, 68]. SpoT possesses a hydrolase activity as well, and therefore, regulates the level of (p)ppGpp and is necessary to terminate the stringent response [67]. In *Lp*, the stringent response, and the RelA and SpoT proteins are necessary for replication inside host cells and initiation of the transmissive phase [67, 68]. Our results clearly show a repression of the translational machinery, cell replication and changes in metabolic processes, which are consistent with the general transcriptional modulations caused by (p)ppGpp [69]. In addition, we have recently shown that the stringent response is required for the survival of *Lp* in water [31].

Due to the imminent shutdown of the translational machinery following water exposure, the important genes should be induced early on to allow the synthesis of their products when *Lp* is still translationally active. Therefore, in order to identify genes essential to maintain long-term survival in water, we examined those that are highly up-regulated within 24 h, a time point when a major metabolic shutdown started to appear. First, many genes associated with the biosynthesis of flagella, including different assembly, regulatory and structural proteins, are up-regulated in *Lp* exposed to water (some are listed in Table 2). This response is comparable to the response of *Lp* that reaches the late replicative phase inside host cells. Due to exploitation of host cell resources and consequent lack of nutrients, the transmissive phase of *Lp* is triggered, leading to the expression of virulence-associated traits such as flagella formation and other factors to promote release from the host [70, 71].

Many genes induced at early time points possess a function related to detoxification and adaptation (Fig. 3). Genes involved in resistance to oxidative stress were induced, including *sodC*, *ahpC* and *ahpD*. We also observed the induction heat shock proteins in water (Table 2). In *E. coli*, heat shock proteins are known to be induced by starvation [72]. They increase bacterial tolerance against various stresses by degrading and reactivating damaged proteins [73]. Therefore, it is plausible that the up-regulation of these genes may help *Lp* to persist in water under starvation conditions. In addition, many genes involved in antibiotic resistance were induced at early time points, including *lpg1492* (spectinomycin phosphotransferase), *lpg1616* (erythromycin transporter), *lpg2151* (aminoglycoside 6-adenylyltransferase), and several efflux pumps (*lpg0257*, *lpg0429*, *lpg1892*, *lpg2189*). Increased resistance to antibiotics is a hallmark of *Lp* MIFs [55]. This phenomenon was also described after incubation of *Lp* in *Acanthamoeba castellanii* buffer at pH 6.5 [74]. Antibiotic resistance genes may be important for *Lp* to compete against antibiotic-producing microorganisms residing in water. In water, *Lp* is more resistant to gentamycin and kanamycin (aminoglycosides) than when it is cultured in rich medium (Fig. 5). Water-exposed *Lp* is also more resistant to erythromycin, but only at a temperature of 37 °C. Since all these antibiotics target the ribosome, it is also possible that the increased resistance is due to a decrease in translational activity, and not solely because of the expression of resistance genes.

Differential expression of some genes involved in virulence was also apparent. Despite the repression of 22 of the 26 structural genes of the Icm/Dot secretion system (Table 1), the expression of many Icm/Dot effectors was increased in water (Table 2). In addition, the enhanced-entry genes *enhA* and *enhB*, and several of the *enhC* homologs, as well as the *rtxA* toxin were induced in water at early time points. On the other hand, the macrophage infectivity potentiator gene, *mip*, was significantly down regulated in water. It is not clear at this point if these changes in expression of virulence genes affect the intracellular multiplication of *Lp* in host cells after incubation in water. We are currently investigating this possibility.

Many genes involved in transcriptional regulation were up-regulated, including numerous transcriptional regulators from different families, DNA binding proteins, a transcription repair coupling factor and the alternative sigma factor σ^54^ (RpoN) (Table 2). Interestingly, RpoN is important for the induction of *fliA* and other flagellar genes [70]. Consistent with previous observations, our results show up-regulation of both σ^54^ and flagellar genes in water.

Several response regulators, sensory proteins and signal transduction proteins involved in “signal transduction/other regulatory functions” were also up-regulated. Among these, many genes encoding proteins harbouring GGDEF/EAL domains were induced in water (some are listed in Table 2). These proteins are involved in the production and destruction of the second messenger c-di-GMP, and several of them have been shown to play a role in the interaction of *Lp* with host cells [75]. Some of them could play a role in sensing exposure to water and regulating the appropriate response. Moreover, expression of the two-component system LetA/S was reduced in water, while expression of the small RNAs RsmY and RsmZ under its control was increased. These sRNAs are necessary to relieve CsrA-mediated repression of transmissive phase traits upon starvation inside host cells [76]. Our results suggest that some of the genes repressed by CsrA could be involved in the survival of *Lp* in water. Interestingly, a link has been made between LetA/S, RpoS and (p)ppGpp [31, 44, 77, 78]. It is noteworthy that expression of the 6S RNA was also induced in water. 6S RNA binds to the RNA polymerase (RNAP) holoenzyme and inhibits its binding to promoters [79]. Since the affinity of 6S RNA to the RNAP depends on sigma factors, 6S RNA can shut down specific transcriptional programs. In *E. coli*, the σ^70^ regulon is turned off in the presence of 6S RNA [80]. In *Lp*, deletion of 6S RNA reduces fitness during intracellular growth [81]. Expression of 6S RNA in water could help *Lp* to switch between different transcriptomic programs, but further investigation is needed to confirm its involvement. There are also many other sRNAs that are differentially regulated, but since their targets are unknown, it is not yet possible to predict specific functions.

Within the highly up-regulated genes in water, we characterized *bdhA*, which is involved in the polyhydroxybutyrate (PHB) cycle [82]. PHB is an important storage polymer in bacteria, which is synthesized as a carbon and energy reserve in the presence of external nutrients and is consumed during starvation [83]. The *bdhA* gene encodes 3-hydroxybutyrate dehydrogenase, which oxidizes depolymerised PHB into acetoacetate and produces reducing power in the form of NADH [84]. Acetoacetate can, then, be further processed into acetyl-CoA, which enters the tricarboxylic acid cycle [84]. *bdhA* is dispensable for *Lp* growth in the presence of external nutrients, as mutations in this gene do not result in any observable growth defects in AYE broth [30]. However, Aurass et al. [85] demonstrated that a *bdhA-patD* mutant strain of *Lp* is defective in breaking down PHB accumulated during growth in a rich medium, resulting in a higher level of cellular PHB than the wild-type. In addition, this mutant has defects in intracellular growth in amoeba and in human macrophages [85]. Therefore, we hypothesize that *bdhA* is important for *Lp* to survive in water, enabling the extraction of carbon and reducing power from PHB in order to allow cell maintenance in the absence of an external energy source. Our results show that the deletion of *bdhA* reduces the survival of *Lp* in water. The Δ*bdhA* strain lost culturability and seems to have entered the VBNC state earlier, resulting in a higher percentage of mortality after 19 weeks of water exposure at 37 °C compared to the wild-type. This survival defect was successfully complemented, supporting the hypothesis that *Lp* needs BdhA for the complete degradation of PHB in order to maintain long-term survival in water. It is noteworthy that the difference in survival between Δ*bdhA* and the wild-type was only observed at 37 °C but not at 25 °C. Since the samples were exhausted after 20 weeks of sampling, we could not determine if the observed difference was eventually be mirrored at 25 °C after a longer period of water exposure. It is possible that survival at 37 °C requires more energy for cell maintenance than at 25 °C because of a higher metabolic rate, protein turnover and overall damage, and thus, the advantage conferred by BdhA is more apparent at the higher temperature.

In addition, we investigated the regulation of *bdhA* expression by RpoS when *Lp* is exposed to water. RpoS is a well-known regulator of the response to nutrient limitation [86]. Recently, we have shown that the stringent response and the sigma factor RpoS are necessary for the survival of *Lp* in water [31]. Based on a GFP reporter assay, we found that *bdhA* is positively regulated by RpoS following exposure to water (Fig. 7). This is consistent with the findings of Hovel-Miner et al. [44], which show that, in a rich medium, *bdhA* in *Lp* is positively regulated by RpoS in the post-exponential phase.

## Conclusions

Our study reveals, for the first time, the global transcriptomic changes of *Lp* in water. Repression of major pathways, such as cell division, transcription and translation, suggests that *Lp* enters a quiescent state in water. The induction of the enhanced-entry genes (*enh*) and some Icm/Dot effectors suggests that *Lp* may be primed to infect a suitable host. Similarly, many genes involved in resistance to antibiotics and oxidative stress, as well as genes involved in the heat shock response were induced. Furthermore, the *bdhA* gene involved in the degradation pathway of the intracellular energy storage compound PHB is highly expressed and positively regulated by RpoS during short-term exposure to water. This gene was found to be important for maintaining long-term survival of *Lp* in water. There is no doubt that many of the genes highly induced upon exposure to water are also necessary for the short and/or long-term survival of *Lp* in water.

## Methods

### Bacterial strains and media

The JR32 strain used in this study is a derivative of *Lp* Philadelphia-1, which is a clinical isolate from the first recognized outbreak of Legionnaires’ disease in 1976 [87]. All *Lp* strains used in this study were derived from JR32 (Table 3). The *rpoS* mutant strain LM1376 was constructed by Hales and Shuman [88]. Unless specified otherwise, *Lp* was cultured on charcoal yeast extract (CYE) agar at 37 °C for 3 days [89]. The media were supplemented with 10 μg/ml gentamycin, 25 μg/ml kanamycin, and/or 1 mM IPTG when appropriate.

**Table 3:** Bacterial strains used in this study

Name	Relevant genotype	Reference
*Legionella pneumophila* strain Phiadelphia-1		
JR32	Sm^R^, r^−^ m^+^	[101]
JR32 pXDC31	JR32 pXDC31, *Ptac*-GFP^+^, Cm^R^	[102]
KS79	JR32 Δ*comR*	[99]
LM1376	JR32 *rpoS*::Tn*903*dGent, Gm^R^	[88]
SPF132	KS79 pXDC39, Cm^R^	This work
SPF194	KS79 Δ*bdhA*, Gm^R^	This work
SPF211	LM1376 pSF53, Gm^R^ Cm^R^	This work
SPF221	LM1376 pSF78, Gm^R^ Cm^R^	This work
SPF236	SPF194 pSF67, Cm^R^	This work
SPF265	JR32 pSF78, Cm^R^	This work
SPF266	JR32 pSF53, Cm^R^	This work
*Escherichia coli*		
DH5α	*supE*44 Δ*lac*U169 (Φ80 *lacZ*ΔM15) *hsdR*17 *recA*1 *endA*1 *gyrA*96 *thi*-1 *relA*1	Invitrogen
pBBR1-MCS5	DH5α, Gm^R^	[103]
pMMB207c	DH5α, pMMB207 Δ*mobA*, Cm^R^	[104]
pXDC39	DH5α, pMMB207c, Δ*Ptac*, Δ*lacI*	Xavier Charpentier
pSF53	DH5α, *PbdhA*-GFP in pXDC39, Cm^R^	This work
pSF67	DH5α, pMMB207c-*bdhA*, Cm^R^	This work
pSF78	DH5α, GFP in pXDC39, Cm^R^	This work

*Escherichia coli* DH5α was used for plasmid construction. *E. coli* was cultured in Luria-Bertani (LB) broth or on LB agar at 37 °C overnight, which were supplemented with 25 μg/ml chloramphenicol when appropriate.

The artificial freshwater medium Fraquil was made with ultra-pure Milli-Q water supplemented with salts and trace metals, at a final concentration of 0.25 μM CaCl_2_, 0.15 μM MgSO_4_, 0.15 μM NaHCO_3_, 10 nM K_2_HPO_4_, 0.1 μM NaNO_3_, 10 nM FeCl_3_, 1 nM CuSO_4_, 0.22 nM (NH_4_)_6_Mo_7_O_24_, 2.5 nM CoCl_2_, 23 nM MnCl_2_, and 4 nM ZnSO_4_ [39].

### Transcriptomic analysis by microarray

*Sample collection.* JR32 was first cultured on CYE plate at 37 °C for 3 days. The colonies were suspended in AYE broth at an OD_600_ of 0.1. Three replicates of this culture were grown under shaking (250 rpm) at 25 °C. Samples for RNA extraction, Live/Dead staining and CFU count were collected from each replicate when the culture reached exponential phase (OD_600_ of 1.0). Then, the remaining culture was centrifuged and washed with Fraquil three times before re-suspending in Fraquil to an OD_600_ of 1.0. Each replicate was then transferred to a BIOSTAT® Q Plus bioreactor vessel (Satorius Stedim Biotech). The temperature was kept constant at 25 °C. Dissolved oxygen was kept constant at around 80 %, by using a stirrer (100 rpm) and injection of air (0.1 bar). Samples for RNA extraction, Live/Dead staining and CFU counts were collected from the vessels after 2, 6 and 24 h of water exposure.

*RNA purification and labelling.* Cells in each sample were pelleted and RNA was extracted using TRIzol reagent (Ambion) according to the manufacturer’s protocol. RNA was then treated with Turbo DNase (Ambion) for 30 min and purified by standard acid phenol extraction [90]. Purified RNA was checked by a NanoDrop® spectrophotometer and PCR to estimate the quantity and quality, as well as to confirm purity. As described by Faucher and Shuman [91], fifteen μg of purified RNA was reverse transcribed into cDNA using random hexamers, aminoallyl dUTP (Invitrogen) and Superscript II reverse transcriptase (Life Sciences) before labeling with Alexa Fluor 647 (Invitrogen). gDNA extracted from JR32 was labelled with Alexa Fluor 546 (Invitrogen) by random priming as described previously [91].

*Microarray design and hybridization*. Gene-specific 50-mer oligonucleotides were designed based on the genome of *Lp* strain Philadelphia-1 using OligoWiz software version 2.2.0 [92, 93]. The probes were designed to hybridize to the center of the target RNAs and prokaryotic settings with default parameters were used. The microarray was produced by photolithography by MYcroarray [94]. Four replicates of each probe as well as the negative and positive probes designed by MYcroarray were included on the DNA microarray. The platform is described in GEO accession number GPL19458. The labelled cDNA and gDNA, used as a reference channel, were hybridized onto the microarray as described previously [91]. The microarray was scanned with an InnoScan microarray scanner (Innopsys) and the data collected was normalized [95, 96]. Statistical analysis between the control (JR32 cultured in AYE broth) and treatments (JR32 exposed to Fraquil for 2, 6 or 24 h) was performed using a paired, one-tailed Student’s *t*-test. The genes with a log_2_ ratio of Treatment/Control >1 or < −1 and *p* < 0.05 were considered differentially expressed. The complete dataset was deposited in GEO (GSE63622).

### RT-qPCR

RNA was extracted and purified from JR32 exposed to AYE broth and Fraquil as described above. Each control or treatment consisted of three biological replicates. One μg of RNA was used for reverse transcription reactions along with a negative control without reverse transcriptase. For qPCR reactions, eleven sets of gene-specific primers were designed with the IDT primer design software [97] (Table 4) and their amplification efficiency were proven to be >85 %. qPCR was performed on an iQ™5 Multicolor Real-Time PCR Detection System (Bio-Rad) using iTaq universal SYBR green supermix (Bio-Rad) according to manufacturer’s protocol. The 16S rRNA gene was used as a reference to normalize the data. Fold change was calculated as described previously [98] and then presented as a log_2_ ratio of Treatment/Control.

**Table 4:** Primer sequences used in this study

**Name**	Purpose	Sequence (5’➔3’)^a^
16 s_QF	16 s rRNA qPCR	AGAGATGCATTAGTGCCTTCGGGA
16 s_QR	16 s rRNA qPCR	ACTAAGGATAAGGGTTGCGCTCGT
25_QF	*lpg0025* qPCR	ATTCCCATCGCCATTTAGAG
25_QR	*lpg0025* qPCR	CAACCCGAGAGGTAACTAATAC
586_QF	*lpg0586* qPCR	GTGGCGTTCCAGTTTGT
586_QR	*lpg0586* qPCR	CTGTCCAGGCAGCATAAC
846_QF	*lpg0846* qPCR	GGTAGAAGGCGATGGTTATC
846_QR	*lpg0846* qPCR	GCCTTCCGGTGGTAATAAA
890_QF	*lpg0890* qPCR	CCTTCCAATCCCATGCTAAAG
890_QR	*lpg0890* qPCR	GTCAAATCCGAGTTCAAGAGG
1206_QF	*lpg1206* qPCR	GCGTCATGAGGATTCTATTCG
1206_QR	*lpg1206* qPCR	GGCCTGTAAATCGTATCAGAC
1284_QF	*lpg1284* qPCR	GTTTATCTCAGAGCGGCAAG
1284_QR	*lpg1284* qPCR	GACATCCTCCAAAGGCTTATC
1659_QF	*lpg1659* qPCR	CGGTCACTCTTTGGTATATGTC
1659_QR	*lpg1659* qPCR	CTGATTGACTGGATCGAACATC
2316_QF	*lpg2316* qPCR	GCCATGTAGCAGAGGAAATC
2316_QR	*lpg2316* qPCR	CTTTATCCACGCCCTGATTG
2487_QF	*lpg2487* qPCR	TCTGTATCTCGGAGCCTATG
2487_QR	*lpg2487* qPCR	GTGGCCTAAACCTGATCTTG
2524_QF	*lpg2524* qPCR	CGCCTGGTATAAAGAACTGC
2524_QR	*lpg2524* qPCR	GAGGCGAAGGTAACCATTTC
bdhA_UpF	Mutant	AGTTCAATACAATCCTTGGTCGC
bdhA_UpR	Mutant	CACGAATTCCTTTTACTATCCTTGTCATTG
bdhA_GnF	Mutant	CGCGAATTCAACGGCATGATGAACCTGAAT
bdhA_GnR	Mutant	CACTCTAGATTAGGTGGCGGTACTTGGGTC
bdhA_DownF	Mutant	CGCTCTAGAACAACCATGACTCGAACTAAAAAATCT
bdhA_DownR	Mutant	CTTTTGAAGACAATTCCGTTTCAT
Com_bdhA_F	Complement	CGCGAGCTCGACAAGGATAGTAAAAGAATGAAACTGAAG
Com_bdhA_R	Complement	CGCTCTAGATCATGGTTGTTTACTCCATGAACC
PromF	Complement	CGTATAATGTGTGGAATTGTGAG
pXDC39-F	GFP assay	GCTTCCACAGCAATGGCATCC
GFP-R	GFP assay	TGTCGACAGGTAATGGTTGTC
GFP_bdhA_F	GFP assay	CGCTCTAGACATAGGGATATCAACCACTACG
GFP_bdhA_R	GFP assay	CGCTCTAGATCTTTTACTATCCTTGTCATTG

^a^The underlined bases indicate different enzyme restriction sites

### Antibiotic resistance test

Cultures of JR32 in AYE broth (OD_600_ of 0.1) were incubated under shaking (250 rpm) at 25 and 37 °C until they reached the exponential phase (OD_600_ of 1.0). For each culture, a 21 ml sample was used for antibiotic exposure and the remainder was centrifuged and washed with Fraquil three times before re-suspending in Fraquil to an OD_600_ of 1.0. The samples were left to incubate in the same condition as before (250 rpm, 25 or 37 °C). After 24 h, a 21 ml sample was again collected from each culture for antibiotic exposure. The procedure of antibiotic exposure was adapted from a previous study [74]. Briefly, one ml aliquots were put in 21 wells (triplicates for control and each of the six treatments) of a 24-well plate (Sarstedt). No antibiotics were added to the control. The six treatments were 100 μg/ml ampicillin, 100 or 500 μg/ml erythromycin, 100 or 500 μg/ml gentamycin and 100 μg/ml kanamycin. The plate was then incubated at 37 °C for 40 min, and the changes in CFU counts between the controls and the treatments were calculated.

### Mutant construction and complementation

For the construction of the *bdhA* deletion mutant, SPF194, 1 kb of the sequences upstream and downstream of *bdhA* were first amplified from KS79 using Taq polymerase (Invitrogen), using the primer sets bdhA_UpF/bdhA_UpR and bdhA_DownF/bdhA_DownR, respectively (Table 4). A gentamycin cassette was amplified from pBBR1-MCS5 using the primer set bdhA_GnF/bdhA_GnR (Table 4). Both bdhA_UpR and bdhA_GnF contain an EcoRI restriction site, while bdhA_GnR and bdhA_DownF contain an XbaI restriction site. All three fragments were digested with EcoRI and/or XbaI (NEB) before ligating with T4 DNA ligase (NEB). The ligation mixture was amplified by PCR using Phusion taq polymerase (NEB) to amplify the 3 kb mutant allele and the purified amplicon was introduced into KS79 through natural transformation [99]. KS79 is constitutively competent due to the lack of *comR*, a negative regulator of competence. The recombinants were selected for gentamycin resistance and successful deletion of *bdhA* was validated by PCR.

For the construction of complemented strain, SPF236, the *bdhA* gene was first amplified from KS79 using primers Com_bdhA_F and Com_bdhA_R, which contain SacI or XbaI restriction sites, respectively. The location of SacI and XbaI restriction site in pMMB207c allowed the *bdhA* gene to be inserted downstream of the P*tac* promoter, allowing the expression of *bdhA* to be induced by IPTG. The amplicon and the pMMB207c plasmid were both digested with SacI and XbaI (NEB) before ligating with T4 DNA ligase (NEB). The ligation mixture was transformed into competent *E. coli* DH5α and the transformants were selected for chloramphenicol resistance. The presence of *bdhA* in the plasmid extracted from transformants was validated by PCR using the primers PromF, which hybridizes to the *Ptac* promoter in pMMB207c, and Com_bdhA_R. Subsequently, this plasmid pSF67 was introduced into SPF194 by electroporation as described by Chen et al. [100] and selected for gentamycin and chloramphenicol resistance before validation by PCR.

### Survival in Fraquil

The wild-type strain KS79, the *bdhA* mutant (SPF194) and the complemented strain (SPF236) were first suspended in Fraquil to an OD_600_ of 0.1. One ml of this culture was mixed with 4 ml of fresh Fraquil, transferred to a 25 cm^2^ plastic flask (Sarstedt) and incubated at 25 or 37 °C. Three replicates were prepared for KS79, Δ*bdhA*, the *bdhA* complement, as well as the *bdhA* complement induced with 1 mM IPTG (Fisher Scientific). The culturability of *Lp* in water was determined weekly by CFU counts on CYE plates. After 19 weeks, the viability was assessed with Live/Dead staining as described below, using KS79 as the control.

### Live/Dead staining

The BacLight™ LIVE/DEAD® bacterial viability kit (Life Technologies) was used to stain the controls and samples according to the manufacturer’s protocol. The Guava easyCyte flow cytometer (EMD Millipore) was used for data acquisition and analysis. Stained Fraquil was used as a blank for instrument setting. Freshly cultured JR32 was used as the live control and JR32 boiled in a water bath for 10 min was used as the dead control for data analysis. Both controls and samples were diluted to an OD_600_ of 0.01 before staining and flow cytometry analysis.

### GFP reporter assay

The JR32 strain carying pXDC31 containing the *Ptac* promoter upstream of the GFP encoding sequence was used as the positive control. For the construction of negative control strains, the plasmids pXDC39 (pMMB207c without *Ptac*) and pXDC31 were first extracted from SPF132 and JR32 pXDC31, respectively, before digesting with SacI and XmnI (NEB). The GFP encoding sequence from pXDC31 was gel purified and ligated with the digested pXDC39 using T4 DNA ligase (NEB). The ligation mixture was transformed into competent *E. coli* DH5α and the transformants were selected for chloramphenicol resistance. The presence of the GFP sequence in the plasmid extracted from transformants was validated by PCR using the primers pXDC39-F and GFP-R. Subsequently, this plasmid, pSF78, was introduced into JR32 and LM1376 (*rpoS* mutant) by electroporation to produce SPF265 and SPF221, respectively. Because of the lack of a promoter upstream of the GFP encoding sequence, these two strains do not express GFP.

For the construction of GFP reporter strains, the 500 bp sequence upstream of *bdhA*, containing the promoter region, was first amplified from KS79 using the primer set GFP_bdhA_F/GFP_bdhA_R with an XbaI restriction site at the 5’ ends. The amplicon and the plasmid pSF78 were digested with XbaI (NEB) and ligated with T4 DNA ligase (NEB). The ligation mixture was transformed into *E. coli* DH5α and the transformants were selected for chlorampheniol resistance. The presence and correct orientation of the inserted promoter in plasmid extracted from transformants were validated by PCR using GFP_bdhA_F and GFP-R. Subsequently, this plasmid, pSF53, was introduced into KS79 or LM1376 by electroporation to produce SPF266 and SPF211, respectively. The location of the XbaI restriction site in pSF78 allowed the promoter region of *bdhA* (*PbdhA*) to be inserted upstream of the GFP encoding sequence, thus the induction of *PbdhA* would result in GFP expression.

For the GFP reporter assay, JR32 pXDC31, SPF265, SPF221, SPF266 as well as SPF211 were suspended in AYE broth at an OD_600_ of 0.1 and in Fraquil at an OD_600_ of 1.0. IPTG (1 mM) was added to the JR32 pXDC31 cultures. All cultures, with three biological replicates each, were incubated at 25 °C and 250 rpm for 24 h, at which point those in AYE broth had reached the exponential phase (OD_600_ of around 1.0). All samples were diluted to an OD_600_ of 0.01 before measuring the green fluorescence signal by flow cytometry.

## Availability of supporting data

The complete microarray data supporting the results of this article is available in the Gene Expression Omnibus repository, [GSE63622; http://www.ncbi.nlm.nih.gov/geo/query/acc.cgi?acc=GSE63622].

## Additional file

Additional file 1:
***Lp*** **genes that are significantly up- or down-regulated in water.** (XLSX 366 kb)

## Abbreviations

AYE: ACES-buffered yeast extract
CFU: Colony-forming units
CYE: Charcoal yeast extract
FSC: Forward scatter
GFP: Green fluorescent protein
HPF: Hibernation promoting factor
IPTG: Isopropyl-β-D-thiogalactopyranoside
LD: Legionnaires’ disease
*Lp*: *Legionella pneumophila*
MIF: Mature intracellular form
PHB: Polyhydroxybutyrate
(p)ppGpp: Guanosine tetraphosphate and guanosine pentaphosphate
RNAP: RNA polymerase
RT-qPCR: Reverse transcription quantitative PCR
T2SS: Type II secretion system
T4BSS: Type IVB secretion system
VBNC: Viable but non-culturable
